# Books Reviewed

**Published:** 1867-05

**Authors:** 


### Books Reviewed.

The Indigestions; or Diseases of the Digestive Organs Functionally Treated. By Thomas King Chambers. Philadelphia: Henry C. Lea.

This volume is upon the symptoms, causes and cure of indigestion, and contains cases related in detail illustrating almost every form of disease in any way connected with indigestion. The author gives us not only his cases, upon which his opinions are based, but also the "distilled essence" of his observation, in his own opinions. When an author withholds his own opinions and only expresses the views of others, he can hardly be supposed to have added anything to the sum of knowledge; happily the author of this work has not fallen into such error, but canvasses the whole ground within the scope of his subject with great accurateness and ability. He says truly: "It is by aid of the digestive viscera alone that consumption can be curable. Medicines addressed to other parts may be indirectly useful sometimes, but they commonly impede the recovery; whereas aid judiciously given in this quarter is always beneficial and often successful."

Quotation is quite unsatisfactory; the whole volume will be read with deep interest by every physician; it treats upon subjects which constantly demand attention from the practitioner, and thus may be called of the greatest practical value.

---

Index of Diseases and their Treatment. By THOMAS HAWKES TANNER. Philadelphia: Lindsay & Blakiston.

As its name indicates this book is an index of diseases. The names of diseases, causes, symptoms and a list of all the remedies ever recommended for cure, are stated in the fewest possible words. Those physicians and students who desire to know but the simplest facts concerning disease and the list of medicines ever proposed as cure, will find exactly what they want in this work. We do not mean that those who are anxious to know as little as possible should make this book a standard, for what there is of it, is unexceptionable. It is valuable as a dictionary, and is to be consulted in the same way and with the same view.

---

Transactions of the Medical Society of the State of Pennsylvania at the Seventeenth Annual Session, held at Wilkesbarre, June, 1866. Fourth Series, part II. Published by the Society.

These transactions contain a number of interesting reports from County Societies relating to the causes which modify the health; the mortality and prevalent diseases; they embody much of practical value to all physicians, but more especially to those of the State. The address delivered by the President, Dr. Wm. Anderson, upon the duties of physicians, is very suggestive, and many would greatly profit by its perusal. To our view the present proceedings may be numbered among the best ever published by the Society, and it is with great satisfaction that we notice such an active participation of the members in the great work of "Medical Union."

---

Reduction of Inverted Uteri by a new method. By THOMAS A. EMMET, M. D., Surgeon in charge of the New York Woman's Hospital, 1866.

Two cases are reported in this monograph by the author, in which he effected the reduction of inverted uteri, by grasping the circumference of the mass as near the seat of inversion as possible, and by upward pressure, the extremities of the fingers acting as a wedge laterally, roll out first that portion last inverted. These manipulations in addition are facilitated in a great measure by the action of the other hand over the abdomen. This method of reduction merits the careful consideration of the profession in all similar cases, in which the uterus is almost entirely contracted to its natural size.

---

Two Cases of Œsophagotomy for the removal of foreign bodies; with a history of the operation. By DAVID W. CHEEVER, M. D. Boston: David Clapp & Son, 1867.

This pamphlet contains a very interesting and instructive account of two operations for œsophagotomy at the Boston City Hospital. The author states that, after a diligent search, he finds a record of only *fifteen* authentic cases, and also shows that as in strangulated hernia, urinary extravasation, or croup, so in œsophagotomy, there is danger in delay. That the risks of operation depend on skill in its performance, and not on its sequences. We believe that more attention should be devoted to this operation, and Dr. Cheever certainly deserves credit in having performed and published the first cases of the kind in this country.

---

Inhalations in the Treatment of Diseases of the Respiratory Passages, particularly as affected by the use of Atomized Fluids. By J. M. Da Costa, M. D. Philadelphia: J. B. Lippincott & Co., 1867.

The substance of this monograph appeared some time since in the numbers of the New York Medical Journal. Attracting considerable attention it has been issued in the present neat and beautiful manner. The readers of this Journal will readily perceive its value by the following index of the chapters, viz:

1. The history of inhalations and the apparatus employed. 2. The mode of administering inhalations. 3. The penetrability of atomized fluids into the air passages. 4. Doses of medicine for inhalation. 5. Therapeutic considerations.

We have not space for an extended review, and as Dr. Da Costa's works are so favorably known, it will not be necessary. We trust this book will find its way to every physician's library.

---

Treatment of Fractures of the Lower Jaw by Interdental Splints. By Thomas Brain Gunning, 1866.

All surgeons have undoubtedly felt more or less the inconvenience arising from the use of the bandage in cases of fracture of the lower jaw, either by their interfering with the circulation, thus preventing union, by their leaving teeth loosened by the injury unsupported, or by their painful restriction of the motions of the jaw, cheeks and lips. The introduction of interdental splints is designed to overcome all these difficulties, and the auther's invention seems eminently adapted to this object.

